# General Analyses of Gene Expression Dependencies on Genetic Burden

**DOI:** 10.3389/fbioe.2020.01017

**Published:** 2020-08-27

**Authors:** Marc González-Colell, Javier Macía

**Affiliations:** Department of Experimental and Health Sciences, Universitat Pompeu Fabra, Barcelona, Spain

**Keywords:** gene expression, gentic circuits, mathematical model, synthetic biological circuits, genetic burden

## Abstract

Over the last decade, the combining of newly developed molecular tools for DNA editing with engineering principles has allowed the creation of complex cellular devices, usually based on complex genetic circuits, for many different purposes. However, when the technological evolution of genetic circuitry is compared with previous technologies such as electronic circuitry, clear limitations regarding the technological scalability of genetic circuitry are observed due to the lack of predictability. To overcome this problem, it is necessary to create new theoretical frameworks for designing genetic circuits in a feasible and reliable manner, taking into account those limitations. Among a number of such limitations, the so-called genetic burden is one of the main constraints. Surprisingly, despite its relevance, little attention has been paid to genetic burden, and it is often not considered when designing genetic circuits. In this study, a new general mathematical formalism is presented, describing the effects of genetic burden on gene expression. The mathematical analysis shows that alterations in gene expression due to genetic burden can be qualitatively described independently of the specific genetic features of the system under consideration. The mathematical model was experimentally tested in different genetic circuits. The experimental evidence coincides with the expected behaviors described by the model in complex scenarios. For instance, observed modulations in the expression levels of constitutive genes in response to changes in the levels of external inducers of gene expression that do not directly modulate them, or the emergence of limitations in gene overexpression, can be understood in terms of genetic burden. The present mathematical formalism provides a useful general framework for gene circuit design that will help to advance synthetic biological systems.

## Introduction

Synthetic biology is a multidisciplinary field of research that seeks to create new biological systems, or to redesign systems that already exist in nature. The achievement of these goals usually requires the introduction of foreign genes into a host cell and their subsequent interconnection, thereby forming genetic circuits that should be designed to work coherently with the rest of the cellular environment. From an engineering point of view, these genetic circuits must be rationally designed according to principles of abstraction, standardization and modularity (Andrianantoandro et al., 2006). Although the use of engineering principles should ensure that the response of genetic devices is predictable, based on the characteristics of its composite parts (Endy, 2005; Canton et al., 2008; Purnick and Weiss, 2009; Brophy and Voigt, 2014; Carbonell-Ballestero et al., 2014; Collins, 2014), in general it is difficult to accurately predict the behavior of a genetic device before its experimental implementation (Cardinale and Arkin, 2012). There are different causes for this degree of unpredictability (Kwok, 2010), which limit the establishment of synthetic biology as an engineering discipline. Among other causes, the so-called metabolic burden (Bailey, 1987; Bentley and Kompala, 1990; Kwok, 2010; de Vos et al., 2011) can alter gene expression and cellular growth rate (Glick, 1995; Scott et al., 2010; Klumpp, 2011; Gyorgy and Del Vecchio, 2014; Mishra et al., 2014; Ceroni et al., 2015; Gyorgy et al., 2015; Carbonell-Ballestero et al., 2016). Gene expression depends on a common pool of cellular resources that must be shared to satisfy genetic demands both to maintain the host cell and to express the foreign genes introduced. In consequence, an increase in the genetic burden can compromise accessibility to cellular resources and may thereby negatively affect the expression of the rest of the genes in the cell.

It is worth mentioning that unlike retroactivity, which occurs when the dynamics of the upstream genes have shown to be disrupted in unexpected ways by a connection of downstream reporter genes with regulatory-protein binding sites that compete with the same binding sites of upstream regulatory (Moriya et al., 2019), competition for cellular resources takes place at the transcriptional and translational levels, involving different mechanisms simultaneously.

In this study we present a new general mathematical formalization and experimental validation describing how expression levels of foreign genes, at steady-state, are modulated by the negative effect associated with their coexistence with the rest of the genes in the cell, i.e., the genetic burden. It is worth mentioning that these negative interactions are always present, independent of the specific nature of the genes considered.

This new formalization has the potential to become a useful tool for designing and predicting the behavior of genetic devices in a reliable manner.

## Mathematical Model of Gene Expression Modulated by Genetic Burden

In order to develop a mathematical formalism describing the dependence between gene expression and genetic burden we departed from the hypothesis that the origin of negative interactions in gene expression due to genetic burden is the limited amount of cellular resources necessary for gene expression. In our approach, we considered a mesoscopic description, in which we classified cellular resources into two sets; resources involved in the transcriptional process and resources involved in translational and post-translational processes.

## Transcriptional Process

Genes can be transcribed in different modes such as in a constitutive manner or following induction by transcription factors. In general, in terms of the gene population, several of these transcriptional modes can coexist; for instance, a given gene can be transcribed both in a constitutive way due to a leaky promoter, and in an induced manner in the presence of a transcription factor. Each of these different modes requires a different amount of transcriptional resources, and therefore they each contribute differently to the total genetic burden supported by the host cell.

**FIGURE 1:**
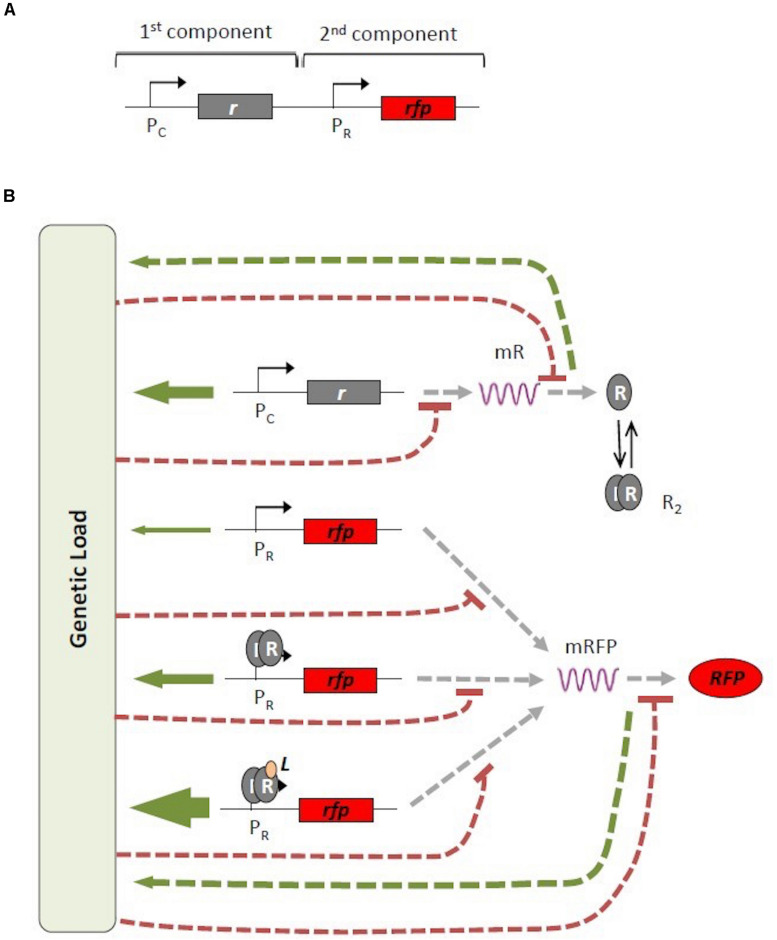
Map of interactions associated with genetic burden. **(A)** Generic architecture of an inducible signal transduction system. The promoter and gene for the receptor protein R [Pc and gray (r) boxes, respectively] and the promoter and gene for the red fluorescent protein [Pr and red (rfp) boxes, respectively] are indicated. **(B)** Different mechanisms or modes of gene expression. Receptor protein *R* is constitutively produced (top row). *RFP* can be produced in three different ways: constitutively (second row); induction by a dimeric complex of the receptor protein *R*_2_ (third row); or induction by a transcriptional complex formed by the dimeric *R*_2_ combined with an external signal *L* (fourth row). All possible transcriptional and translational paths contribute to the total genetic burden with different weights (green arrows), Simultaneously, genetic burden generates a negative effect (dashed red line) on gene transcription and translation. mRFP, messenger RNA for R and RFP.

For illustrative purposes, Figure 1 presents an example of a genetic circuit with different transcriptional modes based on the genetic architecture of the major bacterial signal transduction system for sensing and responding to different environmental conditions in prokaryotic organisms (Stock et al., 2000). The genetic architecture of these systems, which is extensively used in synthetic biology (Fuqua et al., 1994; Williams et al., 2008), involves two genetic components; the first component is the gene for the constitutively expressed receptor protein *R*, that can bind an external signal *L* and subsequently trigger expression of the second gene component, e.g., a gene encoding a fluorescent reporter protein *RFP*. In this architecture, the protein of the second gene component can be expressed according to different modes: in a constitutive manner due to a leaky promoter; induced in response to a dimeric complex (*R*_2_) of the R receptor protein; or induced in response to the transcriptional complex formed by the receptor protein *R*_2_ and the external signal *L* (Carbonell-Ballestero et al., 2014) (see Figure 1). Each transcriptional mode produces a different level of gene transcription with a different associated genetic burden. It should be noted that the total genetic burden is dynamic and changes depending on the abundance of the protein *R* and the ligand *L*. As a consequence, the same genetic construct can exhibit different levels of genetic burden depending on the specific experimental conditions.

To mathematically describe the above model, in general, we can consider the existence of different modes *m*_*k*_ of transcription of a given gene *k*. The rate of gene transcription depends on the concentration of transcriptional resources and on the concentration of other regulators such as transcription factors. According to the law of mass action, these dependencies can be described as:

(1) $$\frac{d\,m\,R\,N\,A_{k}}{d\,t}=\sum_{i=1}^{m_{k}}\beta_{k}^{i}\cdot S_{k}^{i}-\delta_{m\,R\,N\,A_{k}}\cdot m\,R\,N\,A_{k}$$

Here, *mRNA*_*k*_ is the concentration of messenger RNA, and $S_{k}^{i}$ describes the transcriptional complex of gene *k*. The index *i* accounts for all possible transcriptional modes able to produce *mRNA*_*k*_ and $\beta_{k}^{i}$ is a kinetic constant associated with each mode. Finally, δ_*mRNA_k*_ is the degradation rate of *mRNA*_*k*_. In general, $S_{k}^{i}$ depends on two factors, the abundance of free transcriptional resources *S*, shared by all genes, and the concentration of other regulatory elements that are specific for each different transcriptional mode. Defining $g_{k}^{i}\,\left(\omega_{k}^{i}\right)$ as a non-linear function that describes the dependence of gene *k* transcription on the set of regulatory elements {$\omega_{k}^{i}\}$ involved in the transcription mode *i*, at steady-state, $S_{k}^{i}$ can be expressed as:

(2) $$S_{k}^{i}=\lambda_{k}^{i}\cdot g_{k}^{i}\,\left(\omega_{k}^{i}\right)\cdot S$$

where $\lambda_{k}^{i}$ is a kinetic constant and *S* represents the free transcriptional resources, i.e., cellular resources not already involved in gene transcription (see Supplementary Information for details). Because transcriptional cellular resources are limited, we assumed that the total amount of transcriptional resources *S*_*T*_ is constant in a stable cellular culture (Carbonell-Ballestero et al., 2016), i.e.,

(3) $$S_{T}=S+\left\langle S_{C}\right\rangle +\sum_{j=1}^{N}\sum_{i=1}^{m_{j}}S_{j}^{i}$$

Here, ⟨*S*_*C*_⟩ represents the average transcriptional resources devoted to the transcription of genomic genes necessary for host cell. In a first approximation, we assumed that *S*_*c*_ remains constant, on average, over time (Carbonell-Ballestero et al., 2016). Lastly, *N* is the number of foreign genes introduced into the host cell.

At steady-state $\frac{d\,m\,R\,N\,A_{k}}{d\,t}=0$, hence equations (1–3) can be combined to obtain the final concentration of *mRNA*_*k*_ (see Supplementary Information for a complete mathematical derivation)

(4) $$m\,R\,N\,A_{k}=\Upsilon_{k}\cdot \left(\frac{\sum_{i=1}^{m_{k}}\mu_{k}^{i}\cdot g_{k}^{i}\,\left(\omega_{k}^{i}\right)}{1+\sum_{j=1}^{N}\sum_{i=1}^{m_{j}}\lambda_{j}^{i}\cdot g_{j}^{i}\,\left(\omega_{j}^{i}\right)}\right)$$

In this equation, factor *Υ*_*k*_ depends on the total transcriptional resources available to transcribe the *N* foreign genes and on the *mRNA*_*k*_ degradation rate. The numerator accounts for all transcriptional modes involved in the transcription of gene *k*, whereas the denominator accounts for all possible transcriptional modes in the whole set of foreign genes simultaneously expressed in the host cell. It should be noted that terms in the denominator appear when limitation in transcriptional resources is considered. The effect of this limitation is a negative dependence of gene transcription levels on the levels of the rest of the genes present in the host cell, even when these genes are orthogonal, i.e., there is no genetic interaction between them.

## Translational Processes

The synthesis of the final protein *P*_*k*_ encoded by gene *k* needs the interaction between *mRNA*_*k*_ and translational and post-translational cellular resources such as ribosomes. These elements form the translational complex *Q*_*k*_ that is required for protein synthesis. According to the law of mass action law, this protein synthesis can be described as:

(5) $$\frac{d\,P_{k}}{d\,t}=\alpha_{k}\cdot Q_{k}-\delta_{P_{k}}\cdot P_{k}$$

where α_*k*_ is a kinetic constant and δ_*P_k*_ is the protein degradation rate. The translational complex *Q*_*k*_ can be described in terms of the *mRNA*_*k*_ and the free translational resources *Q*:

(6) $$Q_{k}=\epsilon_{k}\cdot m\,R\,N\,A_{k}\cdot Q$$

Here ϵ_*k*_ is a kinetic constant. We assumed that the translational resources *Q*_*T*_ are limited and constant in stable cell culture conditions (Carbonell-Ballestero et al., 2016), hence:

(7) $$Q_{T}=Q+\left\langle Q_{C}\right\rangle +\sum_{j=1}^{N}Q_{j}$$

As previously done, we considered the amount of translational resources devoted to genomic genes to be constant ⟨*Q*_*C*_⟩, on average, over time. At steady-state, i.e., when $\frac{d\,P_{k}}{d\,t}=0$, we can combine equations (5–7) to obtain the final protein concentration (see Supplementary Information for a complete mathematical derivation):

(8) $$P_{k}=\Gamma_{k}\cdot \left(\frac{\sum_{i=1}^{m_{k}}\mu_{k}^{i}\cdot g_{k}^{i}\,\left(\omega_{k}^{i}\right)}{1+\sum_{j=1}^{N}\sum_{i=1}^{m_{j}}\phi_{j}^{i}\cdot \mu_{j}^{i}\cdot g_{j}^{i}\,\left(\omega_{j}^{i}\right)}\right)$$

where $\phi_{j}^{i}$ and $\mu_{j}^{i}$ are kinetic constants. In spite of the fact that the parameters are different, the functional dependence of the translational process, as described in Eq. 8, is similar to the functional dependence of transcriptional process, as described in equation (4). The final protein concentration depends on the genetic mechanism used for protein synthesis, which is described in the numerator, but it is also limited by its own expression and by the expression of the other genes present in the host cell, which is described in the denominator, where the expression of each gene contributes to the total metabolic burden.

## Experimental Model Validation: Case Studies

Equation (8) provides a general description of gene expression levels. This expression depends not only on the specific features of the gene of interest but also on the negative regulation introduced by the genetic burden associated with the expression of the whole set of expressed genes, even when these genes are orthologous. In order to validate our model we considered several case studies in which equation (8) could be applied. In particular, we focused on constitutive and on inducible expression systems present in bacteria based on a receptor protein that binds to an external ligand and triggers the expression of a gene on interest. Toward this goal we built several genetic circuits in *Escherichia coli* (*E. coli*) to assess whether our model properly describes gene expression in different configurations. All genetic constructs used are described in Table S1 and illustrated in Figure S1, and sequences and plasmid maps are provided in the Supplementary Information. Genetic constructs were built using genetic parts form the Parts Registry collection (Registry of Standard Biological Parts, 2020).

## Resource Competition in Constitutive Gene Expression Systems

Our first case study corresponded to a genetic circuit composed of two constitutively expressed genes. For simplicity and for easy quantification of gene expression levels, we analyzed the expression of two different reporter genes, a red fluorescent protein (RFP), and a green fluorescent protein (GFP). In this system, the number of foreign genes (*N*) is 2, there are no external regulators and there is a single mode for gene transcription. According to this description, for the first gene, RFP, we can consider: *m*_*RFP*_ = 1, $\omega_{R\,F\,P}^{1}=\emptyset$ and $g_{R\,F\,P}^{1}\,\left(\emptyset \right)=1$ and, similarly, for the second gene, GFP, we can consider: *m*_*GFP*_ = 1, $\omega_{G\,F\,P}^{1}=\emptyset$ and $g_{G\,F\,P}^{1}\,\left(\emptyset \right)=1$. Applying equation (8) to each gene we get:

(9) $$R\,F\,P=\Gamma_{R\,F\,P}\cdot \left(\frac{\mu_{R\,F\,P}}{1+\phi_{R\,F\,P}\cdot \mu_{R\,F\,P}+\phi_{G\,F\,P}\cdot \mu_{G\,F\,P}}\right)$$

(10) $$G\,F\,P=\Gamma_{G\,F\,P}\cdot \left(\frac{\mu_{G\,F\,P}}{1+\phi_{R\,F\,P}\cdot \mu_{R\,F\,P}+\phi_{G\,F\,P}\cdot \mu_{G\,F\,P}}\right)$$

**FIGURE 2:**
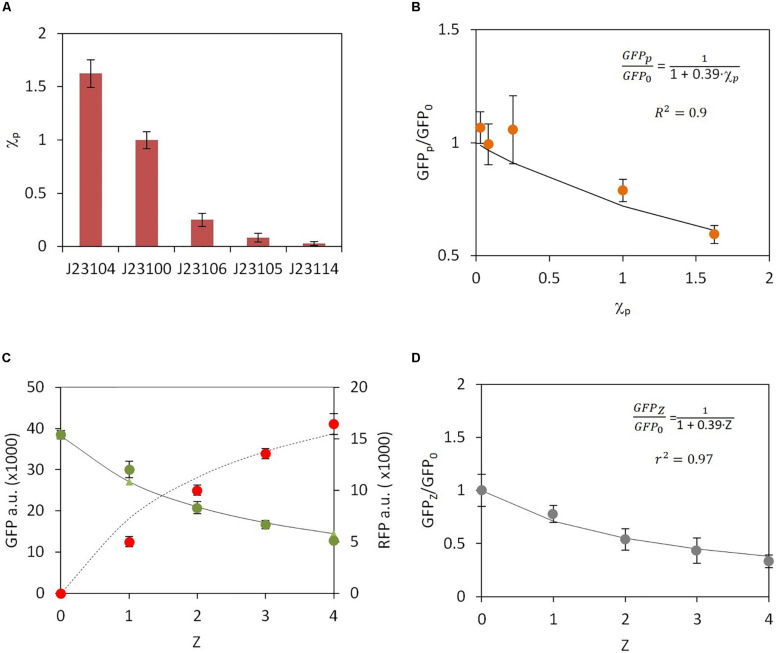
Experimental data and model fitting for constitutive gene expression. **(A)** Experimental measurement of relative promoter activity χ_*p*_. RFP expression was monitored for each promoter. Promoter J23100 was chosen as a reference. **(B)** Change in the ratio of GFP levels in the absence (GFP*_0_*) and upon RFP promoter activity increase (GFP*_*p*_*). Orange dots, experimental data; solid line, model fitting. **(C)** Changes in GFP (green dots) and RFP (red dots) levels, indicated as arbitrary units (a.u.), upon increase in the number of RFP copies, Z. Solid line, GFP model fitting; dashed line, RFP model fitting. **(D)** GFP fold change versus increasing number of RFP copies Z.

Here we simplified the notation according to: $\mu_{R\,F\,P}^{1}\equiv \mu_{R\,F\,P}$, $\mu_{G\,F\,P}^{1}\equiv \mu_{G\,F\,P}$, $\phi_{R\,F\,P}^{1}\equiv \phi_{R\,F\,P}$, and $\phi_{G\,F\,P}^{1}\equiv \phi_{G\,F\,P}$. It should be noted that, whereas the expression of RFP depends on GFP and vice versa, the negative regulation related to genetic burden, described in the denominator of equations (9) and (10), is constant. In order to validate the interdependence of RFP and GFP expression we first used genetic constructs in which the first gene, RFP, was expressed downstream of different promoters, but the second gene, GFP, was always expressed from the same promoter (constructs C1–C5 in Supplementary Table S1, Figure S1). We then analyzed the GFP and RFP levels from the different constructs. The different constitutive promoters used for RFP expression were promoters from Anderson’s collection *J1231xx* (Kelly et al., 2009). However, in all constructs GFP was always constitutively expressed under the *J23100* promoter. Hence, the μ_*RFP*_ values are different for each construct, depending on the specific promoter located upstream of RFP, whereas the value of μ_*GFP*_ is the same in all transformed strains. We first characterized the strength of the different promoters by measuring RFP levels in the absence of GFP (constructs C6-C10 in table S1, Figure S1). The experimental results shown in Figure 2A, allowed direct determination of the relative activity of each promoter with respect to a reference promoter. We chose the promoter *J23100* as the reference. Considering that μ_*RFP*_ directly depends on promoter activity (Kelly et al., 2009), for a given promoter *p* the associated parameter μ_*RFP*_(*p*) can be expressed according to:

(11) $$\mu_{R\,F\,P}\,\left(p\right)=\chi_{p}\cdot \mu_{R\,F\,P}\,\left(J\,23100\right)$$

where the factor χ_*p*_ indicates the relative activity of the promoter *p* with respect to the reference promoter *J23100, i.e.*, *χ*_*J*23100_ = 1Supplementary Table S2 shows the χ_*p*_ values for each promoter relative to that of the J232100. Using equations (9) and (10), after some algebra, the ratio of the constitutive GFP level in the absence of RFP, i.e., GFP_0_, and the GFP level when it is co-expressed with RFP that is expressed from a downstream promoter *p*, i.e., *GFP*_*p*_, can be analytically determined as:

(12) $$\frac{G\,F\,P_{p}}{G\,F\,P_{0}}=\frac{1}{1+\rho \cdot \chi_{p}}$$

With,

(13) $$\rho =\frac{\phi_{R\,F\,P}\cdot \mu_{R\,F\,P}\,\left(J\,23100\right)}{1+\phi_{G\,F\,P}\cdot \mu_{G\,F\,P}}$$

According to this expression, the fold change $\frac{G\,F\,P_{p}}{G\,F\,P_{0}}$ has an inverse dependence on χ_*p*_. Because *GFP*_0_ is independent of χ_*p*_, equation (12) implies that the higher the χ_*p*_ value, the lower the *GFP*_*p*_ value. Figure 2B shows the relationship between the experimentally determined $\frac{G\,F\,P_{p}}{G\,F\,P_{0}}$ and χ_*p*_ compared with the theoretical model. The notable agreement between the experimental results and the theoretical predictions should be noted. However, it is worth mentioning that in Figure 2B construct involving promoter J23105 exhibit higher values of GFP_*p*_/GFP_0_ than the construct based on promoter J23106, despite promoter J23106 has higher activity than J23105 (see Figure 2A). The error bars associated suggest that variability in experimental measures can be the responsible of this deviation from the expected behavior. Best fit was performed for *P* = 0.39 (r^2^ = 0.96). *Matlab R2013a* software was used for fitting the parameters.

The use of an alternative method of increasing RFP expression without changing the promoter sequence was also explored. In this case, levels of RFP were increased by introducing multiple copies of the RFP gene, all of them with the same promoter as that for GFP, *J23100*. Considering *Z* copies of the RFP gene, equation (8) becomes:

(14) $$R\,F\,P\,\left(Z\right)=\Gamma_{R\,F\,P}\cdot \left(\frac{Z\cdot \mu_{R\,F\,P}}{1+Z\cdot \phi_{R\,F\,P}\cdot \mu_{R\,F\,P}+\phi_{G\,F\,P}\cdot \mu_{G\,F\,P}}\right)$$

for RFP and:

(15) $$G\,F\,P\,\left(Z\right)=\Gamma_{R\,F\,P}\cdot \left(\frac{\mu_{G\,F\,P}}{1+Z\cdot \phi_{R\,F\,P}\cdot \mu_{R\,F\,P}+\chi_{G\,F\,P}\cdot \mu_{G\,F\,P}}\right)$$

for GFP. Again, the ratio between the GFP level in the absence of RFP (*Z* = 0), i.e., GFP_0_, and the GFP level in a strain co-expressing *Z* copies of RFP, i.e., GFP_*Z*_, can be theoretically calculated. After some algebra, an expression similar to that described in equation (12) is obtained:

(16) $$\frac{G\,F\,P_{Z}}{G\,F\,P_{0}}=\frac{1}{1+\rho \cdot Z}$$

It should be noted that the parameter *ρ* in equation (16) is the same as that in equation (12). In order to experimentally assess this theoretical relationship between the ratio $\frac{G\,F\,P_{Z}}{G\,F\,P_{0}}$ and the number of copies *Z*, four genetic constructs were built that included from one to four copies of RFP with each copy under the control of the J23100 promoter (constructs C1 and C11-C13 in Supplementary Table S1, Supplementary Figure S1) combined with a single copy of GFP under the J23100 promoter. RFP and GFP levels were then experimentally measured (Figure 2C). These measurements were then compared with the theoretical predictions of GFP dependence on copy number Z that used the previous *ρ* value. The results of the predicted relationship between $\frac{G\,F\,P_{Z}}{G\,F\,P_{0}}$ and *Z* are shown in Figure 2D. A higher *Z* value induces a clear reduction in GFP levels, which reaches up to more than a 50% reduction with respect to levels in the absence of RFP. Moreover, Figure 2C demonstrates that equations (14) and (15) fit with the experimentally measured levels of RFP and GFP respectively. Again, a good agreement between the model predictions and the experimental data was observed, which suggests that the model properly describes the negative effect of genetic burden on orthologous gene expression.

## Variable Genetic Burden in Inducible Gene Expression Systems

The analysis of equation (8) revealed that, in those systems in which gene expression can be modulated by different factors, changes in gene expression correlate with changes in the genetic burden affecting all genes in the host cell. In consequence, the expression of constitutive genes can respond to factors that do not regulate them directly via modifications in genetic burden. Or, for inducible genes, the expression of regulated genes could be modified not only in response to their own regulators but also in response to the regulators of other coexisting genes, exhibiting an unexpected complex multiple regulation.

To experimentally validate these two theoretical scenarios, several genetic constructs were built based on a genetic architecture involving a receptor protein that binds to an external ligand triggering the expression of a gene of interest, similar to the scheme shown in Figure 1. The first genetic device was based on the architecture of the well-known quorum sensing Lux system from *Vibrio fischeri*, which has been extensively used in synthetic biology (Fuqua et al., 1996; Williams et al., 2008). In this system, the receptor protein, termed LuxR, is constitutively expressed and, in the presence of external molecules of 3-oxo-C6-homoserine lactone (C6), the complex LuxR-C6 dimerizes and binds to the Lux promoter (P_*Lux*_), thereby triggering the expression of a downstream gene, e.g., red fluorescent protein (RFP).

In this Lux system three different transcription modes can be identified: (i) basal expression due to the leakiness of P_*Lux*_; (ii) expression induced by the LuxR dimeric complex; and (iii) expression induced by the dimeric LuxR-C6 complex (Carbonell-Ballestero et al., 2014). In order to experimentally confirm these three transcription modes, two genetic constructs were built (constructs C14 and C15 in Supplementary Table S1). The first was composed of RFP under the control of the PLux promoter in the absence of the LuxR protein (Plux-RFP). The second construct combined Plux-RFP with LuxR under the control of the Tet promoter, which behaves as a constitutive promoter in TetR deficient cells (Orth et al., 2000) such as *E. coli* Top10 (see Methods section in Supplementary Information). RFP levels were then measured under the following transcriptional modes: in the absence of LuxR expression, i.e., leaky PLux activity; in the presence of LuxR expression but without C6, i.e., LuxR dimer induction of RFP, and finally in the presence of LuxR expression upon the addition of a high (10^–2^ mM) C6 concentration. The experimental results are shown in Supplementary Figure S2. Despite the fact that all three transcription modes induce RFP expression, there is a clear difference in induced RFP levels between the different transcription modes. Thus the expression of RFP in + LuxR in response to C6 was clearly much higher than that in the other two transcription modes.

The second genetic device built to test our mathematical model of genetic burden, was a construct in which GFP was expressed downstream of the arabinose-inducible promoter pBAD (Khlebnikov et al., 2000) (construct C16 in Supplementary Table S1). In this arabinose-dependent system the protein AraC is the receptor protien and the control of GFP expression by the binding of AraC to pBAD is the second component (see Supplementary Figure S1). In the absence of arabinose, the protein AraC represses the pBAD promoter. In contrast, in the presence of arabinose AraC activates the pBAD promoter, triggering the expression of GFP. As in the Lux system, we can again consider a single transcriptional mode in these arabinose-dependent circuits, i.e., transcription in the presence of arabinose (Supplementary Figure S3).

Even though the mathematical formalism described in this work is a general view, considering *m* potential transcriptional modes, our experimental data indicated that the inducible systems used in our experiments respond mainly to the ligand-based mode (Supplementary Figures S2, S3). The analysis of these systems can therefore be simplified by focusing solely on their ligand-based transcriptional mode.

## Inducible Gene Expression System Coexisting With Constitutively Expressed Genes

The first genetic circuit we analyzed with our mathematical formulation combined constitutive GFP expression under the J23100 promoter with the LuxR inducible system described above controlling RFP expression. This genetic circuit involves three different genes, namely LuxR, RFP and GFP. LuxR and GFP are constitutively expressed, with each of them having a single transcriptional mode. On the other hand, despite the existence of three transcriptional modes for RFP expression, one of these modes (+LuxR, +C6 induction) dominates over the others. According to the mathematical formalization, we can define the set of different regulators for the genes of interest as:

(17) $$\begin{array}{c} \omega_{G\,F\,P}=\left\{\emptyset \right\}\\ \omega_{R\,F\,P}=\left\{L\,u\,x\,R,\text{C}\,6\,\right\} \end{array}$$

It should be noted that, whereas the modulatory function *g*_*GFP*_ = 1 because it corresponds to a constitutive gene expression, the function *g*_*RFP*_(*LuxR*,*C*6) has a complex non-linear dependence with respect to LuxR and C6 due to the genetic mechanisms involving LuxR and C6 (Carbonell-Ballestero et al., 2014). Moreover, an additional layer of complexity exists due to the dependence of LuxR concentration on genetic burden. Fortunately, it is not necessary to consider an explicit description of *g*_*RFP*_(*LuxR*,*C*6) to perform a qualitative analysis of gene expression dependencies. Finally, general gene expression levels described by equation (8) can be reformulated for this particular system as:

(18) $$G\,F\,P=\Gamma_{G\,F\,P}\cdot \left(\frac{\mu_{G\,F\,P}}{\begin{array}{c} 1+\phi_{L\,u\,x\,R}\cdot \mu_{L\,u\,x\,R}+\phi_{G\,F\,P,\cdot }\,\mu_{G\,F\,P}\\ +\phi_{R\,F\,P,\cdot }\,\mu_{R\,F\,P}\cdot g_{R\,F\,P}\,\left(L\,u\,x\,R,C\,6\right) \end{array}}\right)$$

(19) $$R\,F\,P=\Gamma_{R\,F\,P}\cdot \left(\frac{\mu_{R\,F\,P}\cdot g_{R\,F\,P}\,\left(L\,u\,x\,R,C\,6\right)}{\begin{array}{c} 1+\phi_{L\,u\,x\,R}\cdot \mu_{L\,u\,x\,R}+\phi_{G\,F\,P,\cdot }\,\mu_{G\,F\,P}\\ +\phi_{R\,F\,P,\cdot }\,\mu_{R\,F\,P}\cdot g_{R\,F\,P}\,\left(L\,u\,x\,R,C\,6\right) \end{array}}\right)$$

**FIGURE 3:**
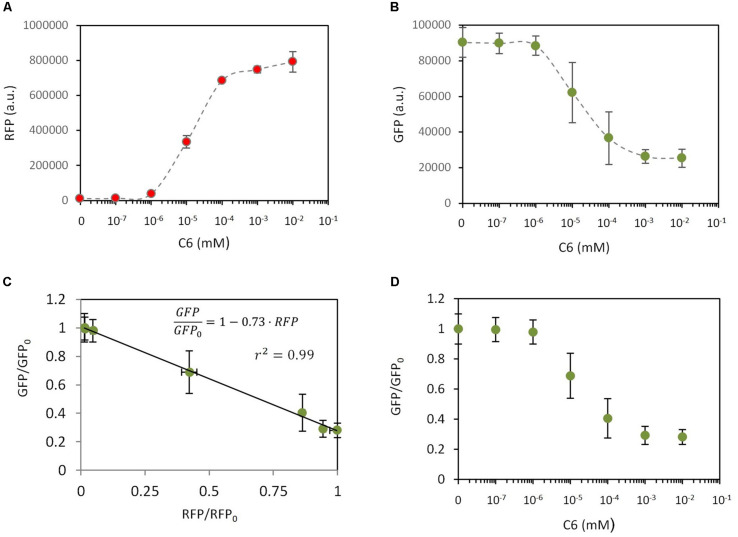
Dependencies of gene expression in response to an external inducer. In all experiments, a construct (C17) from which GFP is constitutively expressed and RFP expression can be induced by C6 addition, was used. RFP levels **(A)** and GFP levels **(B)** were measured (a.u., arbitrary units) in response to different C6 concentrations. **(C)** Correlation between GFP fold change and RFP fold change. Dots are experimental values and the solid line is the model fitting. **(D)** Dependence of GFP fold change on the C6 concentration. Dots are experimental values and the solid line is the model fitting. This fitting combines the experimentally measured relationship between RFP and C6 with the relationship between GFP/GFP_0_ and RFP described by equation (19).

According to this model, RFP levels depend on C6 but GFP also has a significant dependence on C6, despite the fact that GFP is expressed under a constitutive promoter and there is no direct interaction between GFP and C6. To experimentally validate the dependencies described by equations (18) and (19), a new genetic construct was built (construct C17 in Supplementary Table S1, Supplementary Figure S1) that involved a C6-dependent expression of RFP, and constitutive expression of LuxR and GFP. The experimental results regarding RFP and GFP expression levels using this construct and different amounts of C6 are shown in Figure 3. As expected, there was a clear induction of RFP upon increasing the amount of added C6 (Figure 3A). More interesting was the observed dependence of GFP expression on C6 (Figure 3B); thus GFP levels decreased as C6 was increased. Combining equations (18) and (19) it is possible to theoretically determine the GFP fold change. i.e., GFP/GFP_0_ relative to the experimental RFP levels (see Supplementary Information) as:

(20) $$\frac{G\,F\,P}{G\,F\,P_{0}}=1-\frac{\phi_{R\,F\,P}}{\Gamma_{R\,F\,P}}\cdot R\,F\,P$$

Figure 3C shows the experimentally measured relationship between $\frac{G\,F\,P}{G\,F\,P_{0}}$ and RFP and the model fitting. Best fit corresponds to $\frac{\phi_{R\,F\,P}}{\Gamma_{R\,F\,P}}=9.3\cdot 10^{-7}$ (r^2^ = 0.99). Combining equation (20) with the experimental dependence of RFP on C6, it is possible to theoretically correlate $\frac{G\,F\,P}{G\,F\,P_{0}}$ with C6 (Figure 3D). The good agreement between the experimental data and the theoretical description indicated that our model properly captures the effects of metabolic burden in gene expression when an inducible and a constitutive system are combined. This model therefore allows the theoretical determination of the expression levels of a given gene, e.g., GFP, given the experimental levels of a second gene, e.g., RFP.

## System Composed of Two Inducible Genes

The most complex genetic system analyzed in this study was composed of two inducible genes coexisting in the same host cell. Specifically, we analyzed the expression of RFP under a C6-inducible promoter, i.e., the P_*LuxR*_ promoter, and the expression of GFP under the arabinose-dependent pBad promoter. According to the model description, RFP levels would depend not only on C6 concentration but also on arabinose concentration, exhibiting an effective double regulation. Similar behavior would be observed in GFP expression, with a double dependence on arabinose and C6. Moreover, GFP and RFP levels should correlate with each other according to the model description. In this system we consider:

(21) $$\begin{array}{c} \omega_{G\,F\,P}=\left\{A\,r\,a\,C,a\,r\,a\right\}\\ \omega_{R\,F\,P}=\left\{L\,u\,x\,R,C\,6\right\} \end{array}$$

and equation (8) can be applied to both inducible genes:

(22) $$R\,F\,P=\Gamma_{G\,F\,P}\cdot \left(\frac{\mu_{R\,F\,P}\cdot g_{R\,F\,P}\,\left(L\,u\,x\,R,C\,6\right)}{\begin{array}{c} 1+\phi_{L\,u\,x\,R}\cdot \mu_{L\,u\,x\,R}+\phi_{G\,F\,P,\cdot }\,\mu_{G\,F\,P}+\phi_{R\,F\,P,\cdot }\,\mu_{R\,F\,P}\\ \cdot g_{R\,F\,P}\,\left(L\,u\,x\,R,C\,6\right)+\phi_{G\,F\,P,\cdot }\,\mu_{G\,F\,P}\\ \cdot g_{G\,F\,P}\,\left(A\,r\,a\,C,a\,r\,a\right) \end{array}}\right)$$

(23) $$G\,F\,P=\Gamma_{G\,F\,P}\cdot \left(\frac{\mu_{G\,F\,P}\cdot g_{G\,F\,P}\,\left(A\,r\,a\,C,a\,r\,a\right)}{\begin{array}{c} 1+\phi_{L\,u\,x\,R}\cdot \mu_{L\,u\,x\,R}+\phi_{G\,F\,P,\cdot }\,\mu_{G\,F\,P}+\phi_{R\,F\,P,\cdot }\,\mu_{R\,F\,P}\\ \cdot g_{R\,F\,P}\,\left(L\,u\,x\,R,C\,6\right)+\phi_{G\,F\,P,\cdot }\,\mu_{G\,F\,P}\\ \cdot g_{G\,F\,P}\,\left(A\,r\,a\,C,a\,r\,a\right) \end{array}}\right)$$

Here, the modulatory functions *g*_*RFP*_ and *g*_*GFP*_ have a complex non-linear dependence on ω_*RFP*_ and ω_*GFP*_, respectively, not only due to the specific genetic mechanisms (Khlebnikov et al., 2000; Purnick and Weiss, 2009) involved in gene expression but also because LuxR and AraC concentrations can change with genetic burden. Combining equations (21) and (22) it is possible to determine the interdependence between RFP and GFP levels (see Supplementary Information). After some algebra we obtained a linear relationship between GFP fold change and RFP

(24) $$\frac{R\,F\,P}{R\,F\,P_{0}}=1-\frac{\phi_{G\,F\,P}}{\Gamma_{G\,F\,P}}\cdot G\,F\,P$$

**FIGURE 4:**
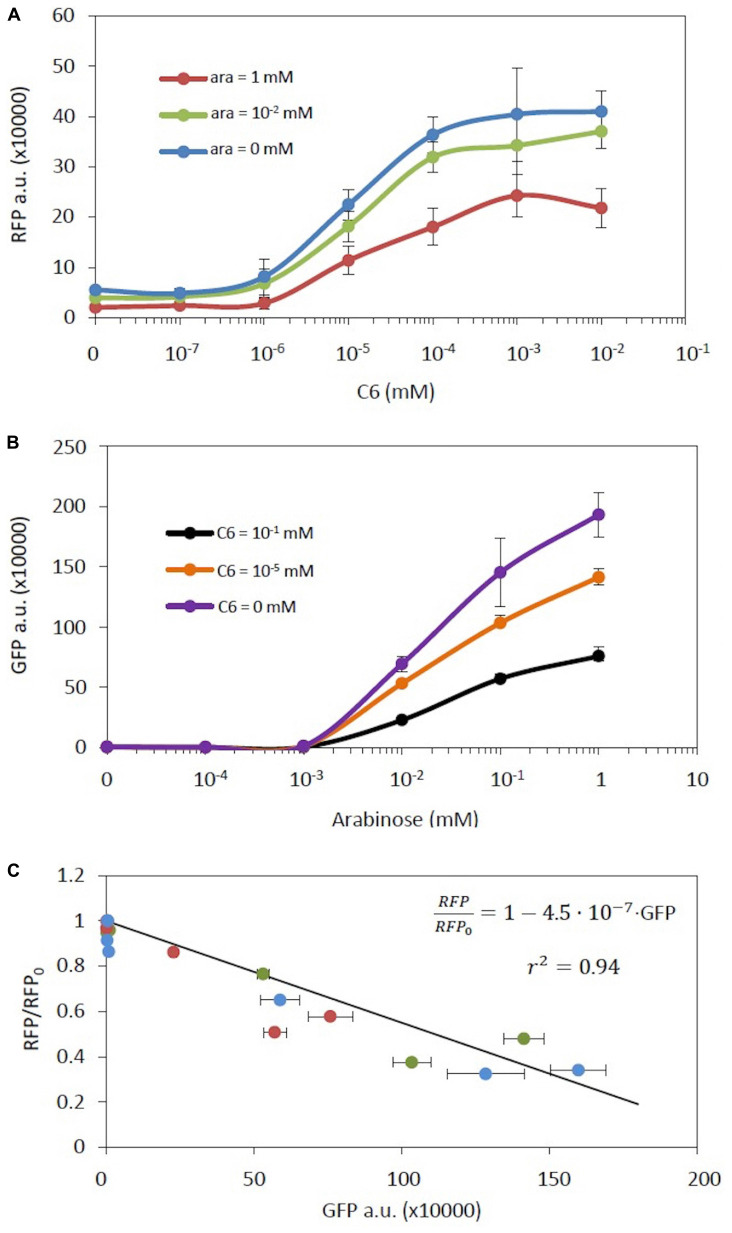
A genetic construct with an inducible RFP and an inducible GFP gene. **(A)** RFP levels vs. the concentration of its inducer C6 at different arabinose concentrations. **(B)** GFP levels vs. the concentration of its inducer arabinose at different C6 concentrations. **(C)** RFP fold-change vs. GFP levels at different C6 concentrations upon different arabinose concentrations. The color code is as in **(A)**. The solid line represents the model fitting. Error bars are the standard deviations of three independent experiments.

This expression is identical to equation (20) despite the fact that in this case both genes are inducible. Furthermore, the slope $\frac{\phi_{G\,F\,P}}{\Gamma_{G\,F\,P}}$ is a constant, independent of inducer concentration. In consequence, while different combinations of C6 and arabinose will produce different levels of RFP and GFP, all of these combinations should maintain the relationship between RFP and GFP described by equation (23). To assess this model we built a new genetic construct composed of these two inducible systems (construct C18 in Supplementary Table S1, Supplementary Figure S1). Figures 4a and 4b show the RFP and GFP levels, respectively, at different concentrations of the combined two inducers. As expected, the expression of each gene has a clear dependence on its own inducer. However, the response of each genetic circuit was strongly affected by the inducer of the other genetic circuit, exhibiting an effective double regulation. Figure 4c shows the relationship between RFP fold change and GFP levels at the indicated concentrations of inducers. It should be noted that all experimental data fit into the same theoretical line described in equation (23), independently of the specific inducer values. Best fit corresponds to $\frac{\phi_{R\,F\,P}}{\Gamma_{R\,F\,P}}=4.5\cdot 10^{-7}$ [r^2^ ( =0.94)].

Based on the above results, genetic burden plays a significant role in gene expression, via an indirect negative regulation. This negative regulation increases with gene expression. In consequence, genetic burden can limit gene overexpression, introducing an upper limit to such expression. To explore this scenario, a genetic construct that combined the constitutive expression of RFP under the J23100 promoter with the inducible expression of the same RFP, based on the LuxR system, was built (construct C19 in Supplementary Table S1, Supplementary Figure S1). *A priori*, the total RFP observed should be the sum of both the constitutive and inducible RFP expression. Equation (8) can be adapted to describe this circuit, considering two different transcriptional modes, the constitutive and the inducible:

(25) $$R\,F\,P=\Gamma_{R\,F\,P}\cdot \left(\frac{\mu_{R\,F\,P}^{1}+\mu_{R\,F\,P}^{2}\cdot g_{R\,F\,P}\,\left(L\,u\,x\,R,C\,6\right)}{\begin{array}{c} 1+\phi_{L\,u\,x\,R}\cdot \mu_{L\,u\,x\,R}+\phi_{R\,F\,P}^{1}\cdot \mu_{R\,F\,P}^{1}\\ +\phi_{R\,F\,P}^{2}\cdot \mu_{R\,F\,P}^{2}\cdot g_{R\,F\,P}\,\left(L\,u\,x\,R,C\,6\right) \end{array}}\right)$$

**FIGURE 5:**
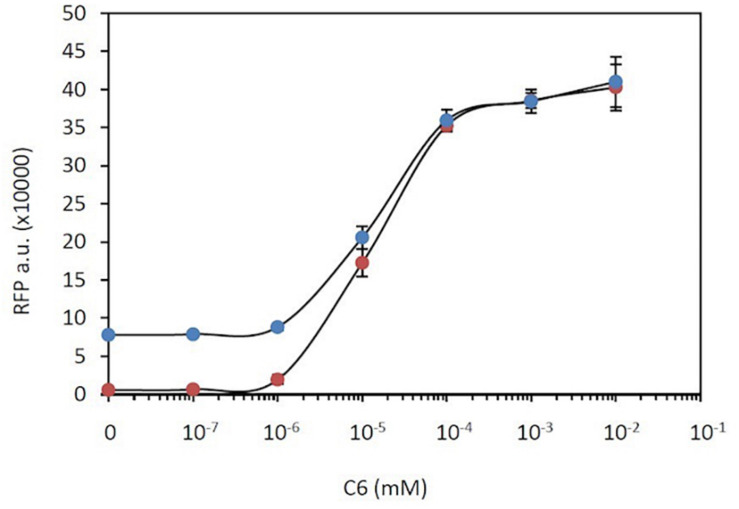
Gene overexpression limitation. RFP expression in a double system that combines constitutive and C6-dependent expression (blue dots) compared with that in a single system with only C6-inducible RFP expression (red dots).

At low induction levels, it is expected that both modes contribute to the total RFP levels. However, at high induction levels, when $\mu_{R\,F\,P}^{2}\cdot g_{R\,F\,P}\,\left(L\,u\,x\,R,C\,6\right)\gg \mu_{R\,F\,P}^{1}$, equation (25) can be approximated to:

$$R\,F\,P≃\Gamma_{R\,F\,P}$$

(26) $$\cdot \left(\frac{\mu_{R\,F\,P}^{2}\cdot g_{R\,F\,P}\,\left(L\,u\,x\,R,C\,6\right)}{1+\phi_{L\,u\,x\,R}\cdot \mu_{L\,u\,x\,R}+\phi_{R\,F\,P}^{2}\cdot \mu_{R\,F\,P}^{2}\cdot g_{R\,F\,P}\,\left(L\,u\,x\,R,C\,6\right)}\right)$$

which is similar to the expression of the inducible construct alone. Figure 5 shows the comparison of experimental measurements of RFP expression in the presence of different concentrations of C6 using construct C19 with both constitutive and inducible RFP versus those when using a genetic construct with only an inducible RFP gene (construct C14). At low induction levels, i.e., C6 < 10^–6^ mM, the main contributor to RFP expression is constitutive expression. At medium induction levels, i.e., 10^–6^ mM < C6 < 10^–4^ mM, although the levels of RFP rise upon induction, the total RFP levels are not the sum of constitutive and inducible contributions but are actually lower. Finally at high induction levels, i.e., C6 > 10^–4^ mM, the RFP expression levels in the double system converge toward those in the single system in which RFP is expressed only upon induction. The lack of additivity, i.e., that the total RFP level is not the sum of the individual contributions of the two systems, is consistent with the theoretical interpretation based on equations (25) and (26). It should be noted that the limitation in RFP overexpression observed in Figure 5 is consistent with the results shown in Figure 2C, in which an increase in the number of copies of RFP is not translated into a linear increase in RFP levels, but the levels are actually lower than expected from such an increase.

## Conclusion

The introduction of foreign genes into a host cell is one of the most widespread methods in synthetic biology, with great potential use in many different fields such as nutrition (Alper et al., 2005), industrial production of valuable molecules (Bayer et al., 2009) or biomedical applications (Ro et al., 2006). However, there are some constraints associated with the introduction of foreign genes into cells (Kwok, 2010). In spite of the fact that genetic burden is one of the most relevant constraints, affecting gene expression at multiple levels, little attention has been paid to its analysis. In this study, we present a new and general mathematical formalism that describes the effects of genetic burden on gene expression. This formalism has the potential to be of interest in the field of synthetic biology by helping to design genetic circuits in a more predictable manner. We have explored the range of applicability of our model in different systems combining: constitutive expression of multiple genes; constitutive and inducible genes; and two inducible genes. In all of these systems, the model correctly describes the experimentally observed behavior. Interestingly, the existence of genetic burden introduces an additional layer of complexity into the genetic circuits because of the emergence of unexpected regulations. For instance, expression of constitutive genes can be *de facto* dependent on external inducers when they coexist with other inducible genes.

Furthermore, theoretical analysis revealed that the overexpression of foreign genes is also limited by genetic burden, pointing out the existence of an upper limit to their expression, which is determined by the availability of cellular resources.

It is worth mentioning that qualitative analysis of gene interdependencies can be performed without an explicit description of genetic interactions described by the functions $g_{k}^{i}$, which points out the general applicability of our mathematical formalism. This fact suggests the existence of general mechanisms associated with limitations in cellular resources that overcome the specific characteristics of each gene. However, future work should be devoted to improve initial model assumptions, such as constancy in genomic genetic burden, which can get better model fitting. Moreover, the possibility of applying this mathematical approximation to more complex systems, determining the limits of its applicability and the potential predictability that these type of models can offer, should be also explored.

## Data Availability Statement

The raw data supporting the conclusions of this article will be made available by the authors, without undue reservation.

## Author Contributions

MG-C and JM designed the project and wrote the manuscript. MG-C performed and analyzed the experiments. JM developed the mathematical model and performed the theoretical analysis. All authors contributed to the article and approved the submitted version.

## Conflict of Interest

The authors declare that the research was conducted in the absence of any commercial or financial relationships that could be construed as a potential conflict of interest.
