# Supplementary material for: General Analyses of Gene Expression Dependencies on Genetic Burden
Source: Front Bioeng Biotechnol. 2020 Aug 27;8:1017. doi: 10.3389/fbioe.2020.01017 (PMC7481379; doi:10.3389/fbioe.2020.01017)
Supplement: Supplementary file 1 [file Table_1.pdf]

Construct	Genetic Structure							
C1	B0014	J23104	B0032	E1010	B0014	J23100	B0032	E0040	B0014
C2	B0014	J23100	B0032	E1010	B0014	J23100	B0032	E0040	B0014
C3	B0014	J23106	B0032	E1010	B0014	J23100	B0032	E0040	B0014
C4	B0014	J23105	B0032	E1010	B0014	J23100	B0032	E0040	B0014
C5	B0014	J23114	B0032	E1010	B0014	J23100	B0032	E0040	B0014
C6	B0014	J23104	B0032	E1010	B0014				
C7	B0014	J23100	B0032	E1010	B0014				
C8	B0014	J23106	B0032	E1010	B0014				
C9	B0014	J23105	B0032	E1010	B0014				
C10	B0014	J23114	B0032	E1010	B0014				
C11	B0014	J23104	2x(B0032 | E1010)	B0014	J23100	B0032	E0040	B0014
C12	B0014	J23104	3x(B0032 | E1010)	B0014	J23100	B0032	E0040	B0014
C13	B0014	J23104	4x(B0032 | E1010)	B0014	J23100	B0032	E0040	B0014
C14	B0014	R0061	B0032	E1010	B0014				
C15	B0014	R0040	B0032	C0062	B0014	R0061	B0032	E1010	B0014
C16	B0014	I0050	B0034	E0040	B0014				
C17	B0014	R0040	B0032	C0062	B0014	R0061	B0032	E1010	B0014
	J23100	B0032	E0040	B0014					
C18	B0014	R0040	B0032	C0062	B0014	R0061	B0032	E1010	B0014
	B0014	I0050	B0034	E0040	B0014				
C19	B0014	R0040	B0032	C0062	B0014	R0061	B0032	E1010	B0014
	J23100	B0032	E1010	B0014					

Table S1. Genetic constructs. Parts were obtained from the Parts Registry collection (http://parts.igem.org). B0014 is a double terminator sequence; J231XX are different promoters from the Anderson collection; B0030, B0032 and B0034 are Ribosomal Binding Sites (RBS); C0062 is the LuxR receptor protein; R0061 is the Lux inducible promoter; I0500 is the AraC protein and the arabinose inducible promoter pBad; E1010 is the red fluorescent protein (RFP); R0040 is the TetR promoter; and E0040 is the green fluorescent protein (GFP).
