# Supplementary material for: General Analyses of Gene Expression Dependencies on Genetic Burden
Source: Front Bioeng Biotechnol. 2020 Aug 27;8:1017. doi: 10.3389/fbioe.2020.01017 (PMC7481379; doi:10.3389/fbioe.2020.01017)
Supplement: Supplementary file 2 [file Table_2.pdf]

**Promoter *p***	**Relative activity χ_p**
**J23104**	**1**
**J23100**	**0.4**
**J23106**	**0.14**
**J23105**	**0.1**
**J23114**	**0.06**

Table S2. Relative activity of different constitutive promoters.
