# Supplementary material for: General Analyses of Gene Expression Dependencies on Genetic Burden
Source: Front Bioeng Biotechnol. 2020 Aug 27;8:1017. doi: 10.3389/fbioe.2020.01017 (PMC7481379; doi:10.3389/fbioe.2020.01017)
Supplement: Supplementary file 3 [file Data_Sheet_1.PDF]

SUPPLEMENTARY INFORMATION

General analyses of gene expression dependencies with genetic load

Marc González-Colell[1], Javier Macía[1,*]

[1]Department of Experimental and Health Sciences. Universitat Pompeu Fabra. Barcelona (Spain)

*Correspondence and requests for materials should be addressed to J.M. (email:javier.macia@upf.edu)

Methods

Strains, media and growth conditions

Cloning and expression experiments were performed in *Escherichia coli* Top10 (Invitrogen, USA). Cells were grown in Lysogeny Broth (LB) at 37 ºC and selected with the appropriate antibiotics (chloramphenicol 35 μg/ml, kanamycin 35 μg/ml; Sigma, USA). Bacterial strains were preserved in LB glycerol 20% (v/v) at −80 ºC. Single colonies obtained from streaked glycerol stocks were inoculated and the cells were grown overnight at 37 ºC with shaking (200 revolutions per minute (rpm)). Overnight cultures were diluted into fresh LB (1/100 dilution) and grown for 5 hours. Diluted cultures were loaded into a 96-well microplate (Nunc, Thermo Fisher Scientific, USA) and induced (see below) in a final volume of 200 μl.

Building of the genetic circuits

Construction of the genetic sensors by cloning was carried out using the Biobrick assembly method and parts from the Spring 2018 iGEM distribution (http://parts.igem.org). All the constructs analysed in this paper were built by combining these parts using 3A assembly. Table S1 shows the genetic structures of the different constructs. Biobrick cloning was performed using an assembly kit (Ginkgo Bioworks, USA). All constructs were included in the Biobricks high copy number plasmid (pSB1AK3) and were transformed using a chemical method. Sanger sequencing confirmed all genetic constructs.

Fluorescence assays for gene expression determination

Colonies of strains containing the plasmid of interest that were obtained from streaked glycerol stocks were grown overnight in LB kanamycin at 37 ºC with continuous shaking. An 100-fold dilution of the overnight culture was grown in fresh LB kanamycin media until the exponential phase, OD_{600} ≈ 0.4. Induction media consisted of LB kanamycin and the appropriate inducer or inducer combination: 3OC6HSL (N-[-ketocaproyl]-L-homoserine lactone (C6); Cayman Chemical Company, USA) and arabinose (L-(+)-Arabinose 98%, Sigma Aldrcih, USA). Different 3OC6HSL concentrations were prepared from an initial stock of $4 \cdot 10^{-2}$ M. Serial dilutions in LB kanamycin, providing final concentrations ranging from 10 µM to 0.001 µM, were prepared the day of the experiment. Different arabinose concentrations were prepared from an initial stock of 0,74 M. Serial dilutions in LB kanamycin, providing final concentrations ranging from 0.1 mM to 0.001 mM were prepared the day of the experiment.

Incubation for *in vivo* measurements was carried out by transferring 2 µl of the diluted cultures and 200 µl of LB kanamycin induction media into a flat bottomed 96-well microplate. LB without cells was also incubated as a background control for both fluorescence and absorbance.

Gene expression induced by a wide range of 3OC6HSL and arabinose concentrations over time was monitored by quantification of the expressed RFP and GFP, respectively. The bacterial cultures were incubated and induced on a Synergy MX microplate reader (BioTek Instruments, USA). Data was acquired at hour 14 in all the experiments, at which time both OD and the fluorescence curves were stabilized and had reached a stationary phase

. Conditions for fluorescence measurements of the RFP were: excitation, 578∓9 nm; and emission, 616∓9 nm, and those for GFP were: excitation, 395∓9 nm; and emission, 509∓9 nm.

Sample (S) absorbance and fluorescence (f) readings (OD_{660} (S) and f (S), respectively) were corrected using respective signal background (B) controls (OD_{660} (B), f (B)). Averaged data were obtained from three independent experiments. Reporter protein Θ was calculated according to the expression:

$$\Theta = \frac{f(S) - f(B)}{OD_{660}(S) - OD_{660}(B)} \qquad (14)$$

Moreover, error bars are the standard deviation from the three independent experiments.

Mathematical model

The mathematical formalization describing the dependence between gene expression and genetic load needs to take into consideration the following assumptions:

i. The number of genes is constant.
ii. The average behaviour of a cell population can be described in a deterministic approach by a set of ordinary differentials equations (ODEs)
iii. Gene expression is measured at the steady state.
iv. The total amount of transcriptional and translational cellular resources remains constant over time

Transcription Process

For a given gene *k*, can be m_k different transcriptional modes to synthetize mRNA. The ratio of mRNA synthesis can be described in terms of the concentration of each different transcriptional complex S_k^i, where index *i* accounts for each transcriptional mode contributing to mRNA synthesis. In general, we can consider the existence m_k different modes allowing the transcription of a given gene *k*. The ratio of gene transcriptions depends on the concentration of transcriptional resources and on the concentration of other regulators, such as transcription factors. According to the law of mass action, these dependences can be described as

$$\frac{dmRNA_k}{dt} = \sum_{i=1}^{m_k} \beta_k^i \cdot S_k^i - \delta_{mRNA_k} \cdot mRNA_k \qquad (A.1)$$

Here $mRNA_k$ is the concentration of RNA messenger and S_k^i describe the transcriptional complex of gene *k*. Index i accounts for all possible transcriptional modes able to produce $mRNA_k$ and β_k^i is a kinetic constant associated to each mode. Finally, δ_{mRNA_k} is the degradation rate of $mRNA_k$. In general, S_k^i depends on two

factors, the abundance of free transcriptional resources *S*, shared by all genes, and the concentration of other regulatory elements, specific for each different transcriptional mode. Defining $g_k^i(\omega_k^i)$ as a non-linear function that describes the dependence of gene *k* transcription with the set of regulatory elements $\{\omega_k^i\}$ involved in the transcription mode i, at steady-state, S_k^i can be expressed as

$$S_k^i = \lambda_k^i \cdot g_k^i(\omega_k^i) \cdot S \qquad \text{(A.2)}$$

where λ_k^i is a kinetic constant and *S* represents the free transcriptional resources, i.e. cellular resources not involved in gene transcription. Hence, equation (A.1) can be written as

$$\frac{dmRNA_k}{dt} = \sum_{i=1}^{m_k} \mu_k^i \cdot g_k^i(\omega_k^i) \cdot S - \delta_{mRNA_k} \cdot mRNA_k \qquad \text{(A.3)}$$

with $\mu_k^i \equiv \beta_k^i \cdot \lambda_k^i$. It is worth mentioning that for those genes that are constitutively expressed, i.e. without other elements modulating gene transcription, $g_k^i(\emptyset) = 1$, which corresponds to a constant ratio formation of transcriptional complex S_k^i. Because transcriptional cellular resources are limited, we assume that the total amount of transcriptional resources S_T is constant in a stable cellular culture, i.e.

$$S_T = S + \langle S_C \rangle + \sum_{j=1}^{N} \sum_{i=1}^{m_j} S_j^i \qquad \text{(A.4)}$$

Here, $\langle S_C \rangle$ is the average transcriptional resources devoted to genomic genes transcription, necessary for host cell maintenance. In a first approximation, we assume that this magnitude remains constant, in average, over time. Lastly, *N* is the number of foreign gene introduced in the host cell.

At steady-state $\frac{dmRNA_k}{dt} = 0$, hence combining equations (A.2) and (A.4) we obtain

$$S_T - \langle S_C \rangle = S + \sum_{j=1}^{N} \sum_{i=1}^{m_j} \lambda_k^i \cdot g_k^i(\omega_k^i) \cdot S \tag{A.5}$$

and

$$S = \frac{S_T - \langle S_C \rangle}{1 + \sum_{j=1}^{N} \sum_{i=1}^{m_j} \lambda_k^i \cdot g_k^i(\omega_k^i)} \tag{A.6}$$

Introducing equation (A.6) in equation (A.3) we get

$$mRNA_k = \Upsilon_k \cdot \left(\frac{\sum_{i=1}^{m_k} \mu_k^i \cdot g_k^i(\omega_k^i)}{1 + \sum_{j=1}^{N} \sum_{i=1}^{m_j} \lambda_j^i \cdot g_j^i(\omega_j^i)} \right) \tag{A.7}$$

with $\Upsilon_k = \frac{S_T - \langle S_C \rangle}{\delta_{mRNA_k}}$.

Translation Process

Synthesis of protein P_k encoded in gene *k*, can be described by

$$\frac{dP_k}{dt} = \alpha_k \cdot Q_k - \delta_{P_k} \cdot P_k \tag{A.8}$$

where Q_k is a translational complex involving $mRNA_k$ molecules and the translational cellular machinery and α_k is a kinetic constant. The concentration of this translational complex can be expressed in terms of $mRNA_k$ concentration and the free translational cellular sources *Q*, i.e.

$$Q_k = \varepsilon_k \cdot mRNA_k \cdot Q \tag{A.9}$$

Here ε_k is a kinetic constant. In consequence

$$\frac{dP_k}{dt} = \alpha_k \cdot \varepsilon_k \cdot mRNA_k \cdot Q - \delta_{P_k} \cdot P_k \tag{A.10}$$

We assume that translational resources Q_T are limited and constant in stable cell culture conditions, hence

$$Q_T = Q + \langle Q_C \rangle + \sum_{t=1}^{N} Q_t \tag{A.11}$$

As previously done, we consider the pull of translational resources devoted to genomic genes $\langle Q_C \rangle$ constant, in average, over time. Hence

$$Q_T = Q + \langle Q_C \rangle + \sum_{t=1}^{N} \varepsilon_t \cdot mRNA_t \cdot Q \tag{A.12}$$

and the free cellular resource *Q* can described by

$$Q = \frac{Q_T - \langle Q_C \rangle}{1 + \sum_{t=1}^{N} \varepsilon_t \cdot mRNA_t} \tag{A.13}$$

Combining equations (A.7) with (A.10) and (A.13) at stead-state, i.e. $\frac{dP_k}{dt} = 0$, we obtain

$$P_k = \frac{\alpha_k \cdot \varepsilon_k \cdot \Upsilon_k}{\delta_{P_k}} \cdot \left(\frac{\sum_{i=1}^{m_k} \mu_k^i \cdot g_k^i(\omega_k^i)}{1 + \sum_{j=1}^{N} \sum_{i=1}^{m_j} \lambda_j^i \cdot g_j^i(\omega_j^i)} \right) \cdot \frac{Q_T - \langle Q_C \rangle}{1 + \sum_{t=1}^{N} \varepsilon_t \cdot \Upsilon_t \cdot \left(\frac{\sum_{i=1}^{m_t} \mu_t^i \cdot g_t^i(\omega_t^i)}{1 + \sum_{j=1}^{N} \sum_{i=1}^{m_j} \lambda_j^i \cdot g_j^i(\omega_j^i)} \right)} \tag{A.14}$$

Simplifying, we obtain the final expression

$$P_k = \Gamma_k \cdot \left(\frac{\sum_{i=1}^{m_k} \mu_k^i \cdot g_k^i(\omega_k^i)}{1 + \sum_{t=1}^{N} \sum_{i=1}^{m_t} \phi_t^i \cdot \mu_t^i \cdot g_t^i(\omega_t^i)} \right) \tag{A.15}$$

with

$$\Gamma_k \equiv \frac{\alpha_k \cdot \varepsilon_k \cdot \Upsilon_k \cdot (Q_T - \langle Q_C \rangle)}{\delta_{P_k}} \tag{A.16}$$

and

$$\phi_t^i \equiv \left(\frac{\lambda_t^i}{\mu_t^i} + \varepsilon_t \cdot \Upsilon_t \right) \tag{A.17}$$

Interdependence between gene expression in genetic construct composed by a constitute gene coexisting with a two-component inducible system.

Considering a general genetic construct composed by an inducible gene G_I, based on two-component architecture, coexisting with another gene constitutively expressed G_C, general equation (A.15) can be applied

$$G_C = \Gamma_{G_C} \cdot \left(\frac{\mu_{G_C}}{1 + \phi_R \cdot \mu_R + \phi_{G_C} \mu_{G_C} + \phi_{G_I} \cdot \mu_{G_I} \cdot g_{G_I}(R, I)} \right) \quad \text{(A.18)}$$

$$G_I = \Gamma_{G_I} \cdot \left(\frac{\mu_{G_I} \cdot g_{G_I}(R, I)}{1 + \phi_R \cdot \mu_R + \phi_{G_C} \mu_{G_C} + \phi_{G_I} \cdot \mu_{G_I} \cdot g_{G_I}(R, I)} \right) \quad \text{(A.19)}$$

Here, R is the receptor protein, necessary in the two-component architecture, and I is the external inducer. Function $g_{G_I}(R, I)$ describes the transcription rate of gene G_I, which depends on R and I concentrations. Moreover, concentration of R, despite is constitutively expressed, depends on the expression levels of the other genes via genetic load. In consequence, function $g_{G_I}(R, I)$ has a complex dependence with R and I. In order to determine the dependence of G_C fold change with respect to inducer concentration we define the expression of G_C in absence of inducer, i.e. I=0, as

$$G_C^0 = \Gamma_{G_C} \cdot \left(\frac{\mu_{G_C}}{1 + \phi_R \cdot \mu_R + \phi_{G_C} \mu_{G_C}} \right) \quad \text{(A.20)}$$

Hence, the fold change

$$\frac{G_C}{G_C^0} = \left(\frac{1 + \phi_R \cdot \mu_R + \phi_{G_C} \cdot \mu_{G_C}}{1 + \phi_R \cdot \mu_R + \phi_{G_C} \mu_{G_C} + \phi_{G_I} \cdot \mu_{G_I} \cdot g_{G_I}(R, I)} \right) \quad \text{(A.21)}$$

On the other hand, $g_{G_I}(R, I)$, despite its complexity, can be described in terms of G_I expression levels using equation (A.19)

$$g_{G_I}(R, I) = \frac{\left(1 + \phi_{G_C} \cdot \mu_{G_C} + \phi_R \cdot \mu_R\right) \cdot G_I}{\mu_{G_I} \cdot \left(\Gamma_{G_I} - \phi_{G_I} \cdot G_I\right)} \quad \text{(A.22)}$$

Introducing (A.22) in (A.21) we obtain

$$\frac{G_C}{G_C^0} = \left(\frac{1 + \phi_R \cdot \mu_R + \phi_{G_C} \cdot \mu_{G_C}}{1 + \phi_R \cdot \mu_R + \phi_{G_C} \mu_{G_C} + \phi_{G_I} \cdot \mu_{G_I} \cdot \left[\frac{\left(1 + \phi_{G_C} \cdot \mu_{G_C} + \phi_R \cdot \mu_R\right) \cdot G_I}{\mu_{G_I} \cdot \left(\Gamma_{G_I} - \phi_{G_I} \cdot G_I\right)} \right]} \right) \tag{A.23}$$

After some algebra, the final relationship is obtained

$$\frac{G_C}{G_C^0} = 1 - \frac{\phi_{G_I}}{\Gamma_{G_I}} \cdot G_I \tag{A.24}$$

Interdependence between gene expressions in genetic constructs composed by two inducible genes based on two-component architectures.

Genetic constructs composed of two inducible genes based on two-component architecture involve four different genes, namely the two receptor proteins R_1 and R_2 that in response to external induces I_1 and I_2 trigger the expression of genes G_1 and G_2. Applying equation (A.15) to this system

$$G_1 = \Gamma_{G_1} \cdot \left(\frac{\mu_{G_1} \cdot g_{G_1}(R_1, I_1)}{1 + \phi_{R_1} \cdot \mu_{R_1} + \phi_{R_2} \cdot \mu_{R_2} + \phi_{G_1} \cdot \mu_{G_1} \cdot g_{G_1}(R_1, I_1) + \phi_{G_2} \cdot \mu_{G_2} \cdot g_{G_2}(R_2, I_2)} \right) \tag{A.25}$$

$$G_2 = \Gamma_{G_2} \cdot \left(\frac{\mu_{G_2} \cdot g_{G_2}(R_2, I_2)}{1 + \phi_{R_1} \cdot \mu_{R_1} + \phi_{R_2} \cdot \mu_{R_2} + \phi_{G_1} \cdot \mu_{G_1} \cdot g_{G_1}(R_1, I_1) + \phi_{G_2} \cdot \mu_{G_2} \cdot g_{G_2}(R_2, I_2)} \right) \tag{A.26}$$

From these equations it is possible to determine the fold change in one of the genes with respect the levels of expression of the other gene, depending on its inducer concentration.

In absence of the second inducer I_2, the expression levels of the first gene G_1 are

$$G_1^0 = \Gamma_{G_1} \cdot \left(\frac{\mu_{G_1} \cdot g_{G_1}(R_1, I_1)}{1 + \phi_{R_1} \cdot \mu_{R_1} + \phi_{R_2} \cdot \mu_{R_2} + \phi_{G_1} \cdot \mu_{G_1} \cdot g_{G_1}(R_1, I_1)} \right) \tag{A.27}$$

and the fold change

$$\frac{G_1}{G_1^0} = \left(\frac{1 + \phi_{R_1} \cdot \mu_{R_1} + \phi_{R_2} \cdot \mu_{R_2} + \phi_{G_1} \cdot \mu_{G_1} \cdot g_{G_1}(R_1, I_1)}{1 + \phi_{R_1} \cdot \mu_{R_1} + \phi_{R_2} \cdot \mu_{R_2} + \phi_{G_1} \cdot \mu_{G_1} \cdot g_{G_1}(R_1, I_1) + \phi_{G_2} \cdot \mu_{G_2} \cdot g_{G_2}(R_2, I_2)}\right) \quad \text{(A.28)}$$

From equation (A.26) we can express the modulatory function $g_{G_2}(R_2, I_2)$ in terms of

$$g_{G_2}(R_2, I_2) = \frac{\left(1 + \phi_{R_1} \cdot \mu_{R_1} + \phi_{R_2} \cdot \mu_{R_2} + \phi_{G_1} \cdot \mu_{G_1} \cdot g_{G_1}(R_1, I_1)\right) \cdot G_2}{\mu_{G_2} \cdot \left(\Gamma_{G_2} - \phi_{G_2} \cdot G_2\right)} \quad \text{(A.29)}$$

Finally, introducing equation (A.29) in (A.28) and simplifying the final expression is obtained

$$\frac{G_1}{G_1^0} = 1 - \frac{\phi_{G_2}}{\Gamma_{G_2}} \cdot G_2 \quad \text{(A.30)}$$
