# Supplementary material for: General Analyses of Gene Expression Dependencies on Genetic Burden
Source: Front Bioeng Biotechnol. 2020 Aug 27;8:1017. doi: 10.3389/fbioe.2020.01017 (PMC7481379; doi:10.3389/fbioe.2020.01017)
Supplement: Supplementary file 4 [file Image_1.pdf]

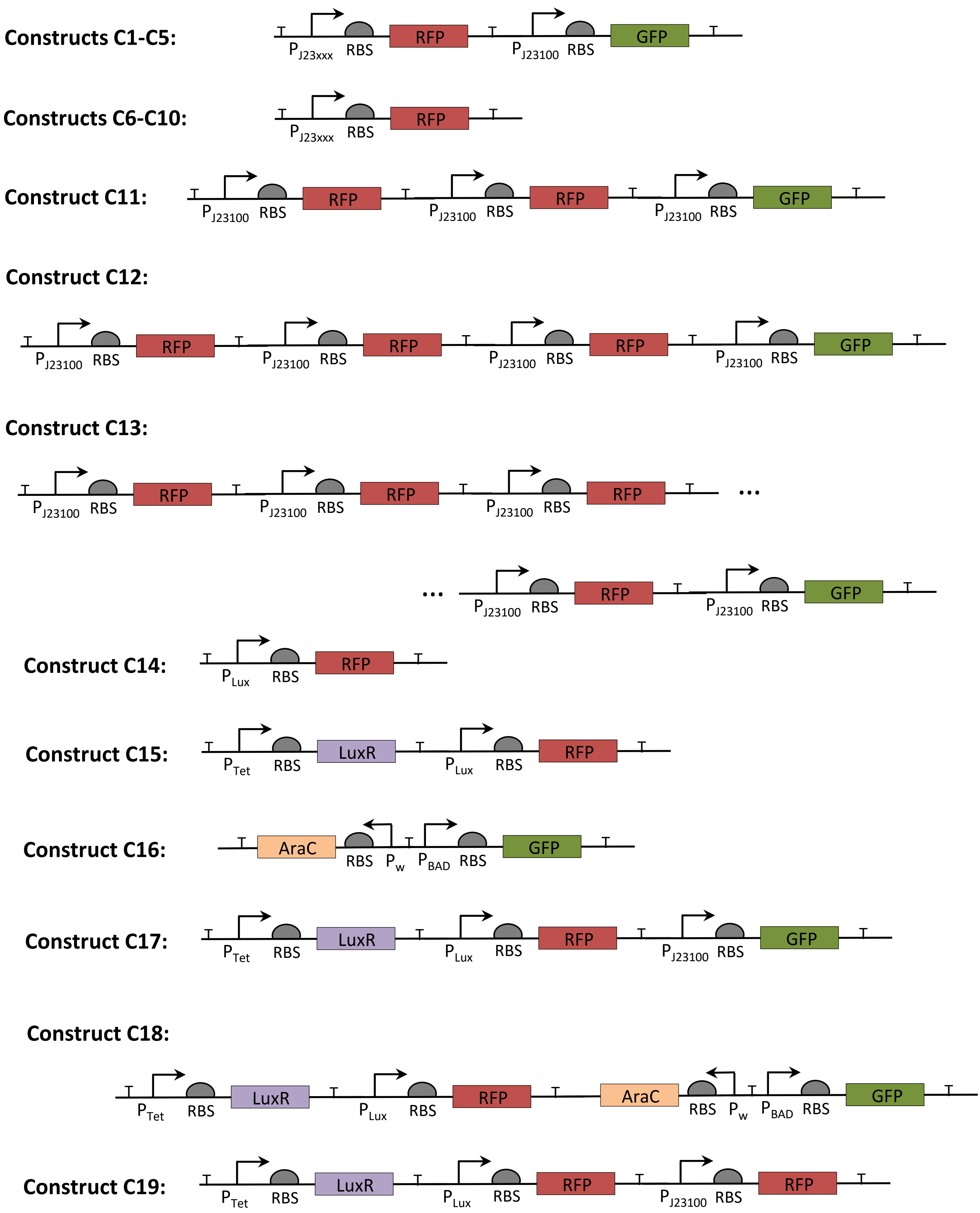

Fig. S1. Schematic representation of the genetic constructs analysed in this study.

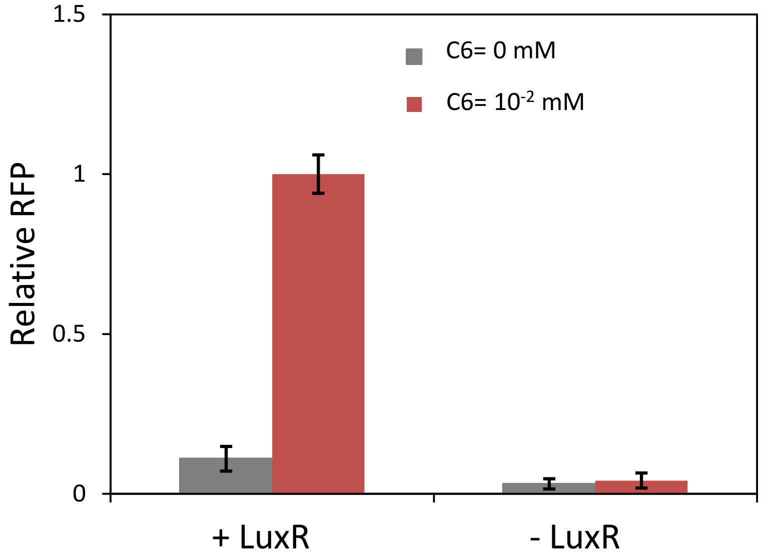

Fig. S2. Relative RFP levels in the different transcriptional modes in the LuxR system. RFP levels in the presence (+ LuxR) or absence (- LuxR) of LuxR, in the presence (grey bars) or absence (red bars) of 10^{-2} mM C6. Error barsc are the standard deviation from three independent experiments. RFP levels are shown relative to those in + LuxR,+C6.

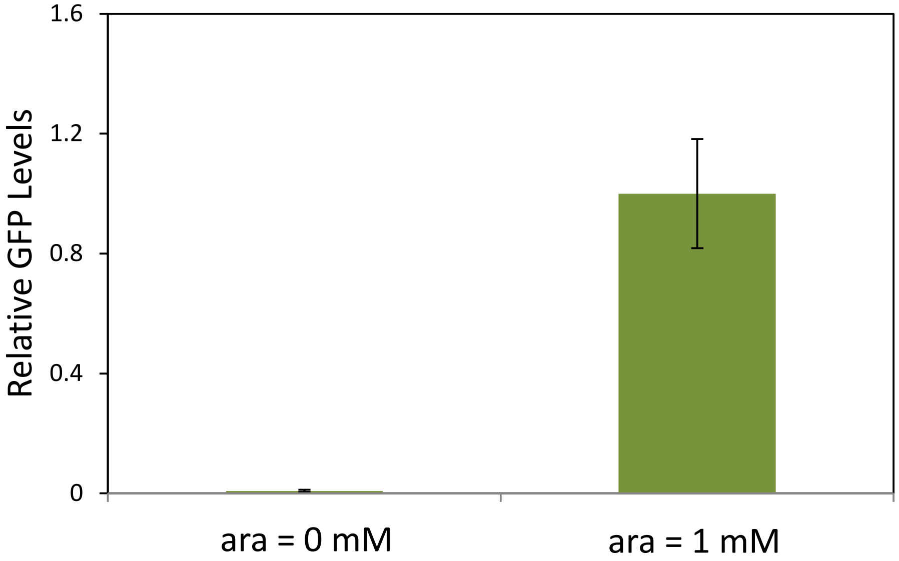

Fig. S3. Relative GFP levels in the arabinose-inducible system. Relative GFP levels in the presence or absence of arabinose (ara).
